# European guidelines on peri-operative venous thromboembolism prophylaxis: first update. Chapter 12: Urology

**DOI:** 10.1097/EJA.0000000000002012

**Published:** 2024-07-10

**Authors:** Kari A.O. Tikkinen, Rufus Cartwright, Anders Gadegaard Jensen, Philippe D. Violette

**Affiliations:** From the Department of Urology, University of Helsinki and Helsinki University Hospital, Helsinki (KAOT), Department of Surgery, South Karelian Central Hospital, Lappeenranta, Finland (KAOT), Department of Health Research Methods, Evidence and Impact, McMaster University, Hamilton, Ontario, Canada (KAOT, PDV), Departments of Gynaecology and Gender Affirmation Surgery, Chelsea and Westminster NHS Foundation Trust (RC), Department of Epidemiology and Biostatistics, Imperial College London, London, UK (RC), Department of Anesthesiology, Odense University Hospital, Odense, Denmark (AGJ), Department of Surgery, Woodstock Hospital, Woodstock, Ontario, Canada (PDV) and European Association of Urology (EAU), Arnhem, The Netherlands (KAOT, RC, PDV)

## Introduction

The European Society of Anaesthesiology and Intensive Care (ESAIC) thromboprophylaxis guideline convened a panel comprising urologists, urogynaecologists, anaesthesiologists and experienced guideline methodologists to provide a summary guideline for the use of thromboprophylaxis in urology. The panel adhered to the GRADE approach in elaborating three guideline statements intended to summarise when thromboprophylaxis clearly should not be used, clearly should be used and when a more tailored approach is preferred. The panel also provided an overview of the principles of when to start prophylaxis, optimal duration of prophylaxis and preferred agents for urological procedures.

## Recommendation 1

(1) In all patients undergoing ambulatory day surgery (e.g. circumcision, vasectomy, hydrocoelectomy and ureteroscopy), the Panel recommends against use of pharmacological prophylaxis (Grade 1B), and against use of mechanical prophylaxis (Grade 1B).

*Rationale*: The Panel understands the risk of symptomatic venous thromboembolism (VTE) in this group to be similar to the risk of VTE in the general population. Administering pharmacological (or mechanical) thromboprophylaxis in this context would result in only marginal reductions in VTE risk, while potentially increasing the likelihood of bleeding complications, overall healthcare expenses and/or the burden of patient care.^1,2^

## Recommendation 2

(1) In all patients undergoing open radical cystectomy, or open radical prostatectomy with extended lymphadenectomy, the Panel recommends use of pharmacological prophylaxis (Grade 1A or Grade 1B, depending on risk stratum), and suggests use of mechanical prophylaxis (Grade 2C).

*Rationale*: These procedures have a relatively high risk of VTE with much lower risk of major bleeding. Procedural factors for these procedures outweigh patient-specific factors in determining the overall net benefit as net benefit remains in favour of prophylaxis across all strata of patient risk. The Panel believes that for all patients the net benefit is sufficient to justify the additional burden and cost of using extended pharmacological thromboprophylaxis and may justify the use of mechanical prophylaxis until ambulation.^1–3^

## Recommendation 3

(1) In patients undergoing most other urological procedures, the risk prediction varies by surgical procedure and patient factors and a more detailed approach is preferred.

*Rationale:* For most urological surgeries, procedure and patient-specific factors can sway the balance of VTE and bleeding risk such that net benefit can vary for a given procedure. The primary procedure-specific factors that can affect VTE and bleeding are the procedure itself, the surgical approach (laparoscopic, open, robotic), and the extent of lymphadenectomy. The primary patient factors that affect VTE risk include prior history of VTE, age and BMI. Consequently, recommendations can also vary for a given urological procedure.^3–5^

Illustration: To illustrate how net benefit can vary with procedure and patient-specific factors, consider the use of extended pharmacological prophylaxis for patients undergoing robotic-assisted radical prostatectomy (Table 1). In this table, we can see that patients at low risk of VTE with any degree of lymphadenectomy probably do not benefit from pharmacological prophylaxis. However, for patients at medium or high risk of thrombosis, the benefit of (extended) prophylaxis varies depending on degree of lymph node dissection. In this way, the preferred approach is to tailor the recommendation to specific clinical circumstances rather than attempt to make one recommendation for all patients undergoing robotic-assisted radical prostatectomy. This approach has been elaborated for a large number of urological procedures in the European Association of Urology guideline on Thromboprophylaxis in Urological Surgery.^3^

**Table 1:** Risk stratification among patients undergoing robotic-assisted radical prostatectomy

Procedure	Risk of VTE stratified by patient risk category^a^ (per 1000 patients)	Risk of major bleed requiring re-intervention (per 1000 patients)	Net benefit with prophylaxis^b^ (per 1000 patients)	Recommendation based on net benefit where: ≥10, strong for; 5 to 10, weak for; 1 to 5, weak against; <1, strong against
Robotic prostatectomy without pelvic lymphadenectomy (PLND)	Low: 2.0 Medium: 5.0 High risk: 9.0	4.0	−1.1 0.4 2.4	Strong against Strong against Weak for
Robotic with standard PLND	Low risk: 5.0 Medium risk: 9.0 High risk: 19	6.0	−0.7 1.3 6.3	Strong against Weak for Weak for
Robotic with extended PLND	Low risk: 9.0 Medium risk: 19 High risk: 37	8.0	0.3 5.3 14	Strong against Weak for Strong for

VTE, venous thromboembolism.

a In the VTE risk strata, patients with no VTE risk factor are classified as low VTE risk, patients with one VTE risk factor (age 75 or more; or BMI of 35 or more) as medium VTE risk, and patients with two risk factors and those with personal history of VTE as high VTE risk.

b Net benefit is equal to absolute reduction in VTE risk minus absolute increase in bleeding risk (with twice the weight for major bleeding as for VTE). Net benefit with prophylaxis assumes an approximately 50% decrease in VTE and an approximately 50% increase in major bleed based on meta-analysis by Gould *et al.*^5^ and updated by Lavikainen *et al.*^6^

### Principle 1

(1) The Panel recommends starting thromboprophylaxis on post operative day 1 compared with presurgery.

*Rationale:* Most major bleeding events occur near to the time of surgery, with more than 40% of major bleeding events on the day of surgery,^7^ whereas VTE occur most frequently during the first postoperative week and continue to occur substantially up to 1 month postoperatively.^8^ A meta-analysis of randomised trials did not find important extra benefit or harm between groups receiving the initial dose of pharmacologic thromboprophylaxis at different times preoperatively or postoperatively.^9^ In this context, the panel suggests initiating administration of pharmacologic thromboprophylaxis beginning the day after surgery.

### Principle 2

(1) The Panel recommends continuing thromboprophylaxis for an extended period of 2 to 4 weeks when pharmacological thromboprophylaxis is used.

*Rationale:* Moderate certainty evidence has shown that 47% of symptomatic VTE in first 4 weeks postsurgery occur during the first week followed by an additional 27% during the second, 16% during the third, and 10% during the fourth week postsurgery.^9^ In contrast, of the cumulative risk during the first 4 weeks postsurgery, more than 40% of major bleeds occur within 24 h of surgery and approximately 80% during the first week postsurgery.^7^ Randomised trials have shown that extended pharmacological thromboprophylaxis provides a significant reduction in the risk of VTE compared with thromboprophylaxis during hospital admittance only, without increasing bleeding complications.^10^ A more precise ‘optimal’ duration of prophylaxis will be additionally influenced by baseline risk, cost of agent, burden of care and patient values and preferences.

### Principle 3

(1) The Panel recommends that low-molecular-weight heparin (LMWH) can be used and suggests that direct oral anticoagulants (DOAC) may be used for pharmacological thromboprophylaxis.

*Explanation:* High-quality evidence found that LMWHs significantly reduce the risk of VTE compared with no anticoagulation.^2,6,11^ In the absence of direct comparisons between LMWH and DOAC, and between DOAC and no anticoagulation among patients undergoing urological surgery, evidence from a systematic review and network meta-analysis suggests the efficacy and safety of DOACs is comparable to LMWH.^11^ Some caution may be considered among postoperative patients who are expected to have decreased gastric motility or ileus.
